# Novel *TNFAIP3* microdeletion in a girl with infantile-onset inflammatory bowel disease complicated by a severe perianal lesion

**DOI:** 10.1038/s41439-020-00128-4

**Published:** 2021-01-14

**Authors:** Kosuke Taniguchi, Mikihiro Inoue, Katsuhiro Arai, Keiichi Uchida, Osuke Migita, Yui Akemoto, Junya Hirayama, Ichiro Takeuchi, Hirotaka Shimizu, Kenichiro Hata

**Affiliations:** 1 Department of Maternal-Fetal Biology, National Research Institute for Child Health and Development, Tokyo, 157-8535 Japan; 2 Department of Gastrointestinal and Pediatric Surgery, Mie University Graduate School of Medicine, Tsu, Mie 514-8507 Japan; 3 Division of Gastroenterology, National Center for Child Health and Development, Tokyo, 157-8535 Japan; 4 Department of Pediatrics, St. Marianna University School of Medicine, Kanagawa, 216-8511 Japan; 5 Department of Anatomic Pathology, Hirosaki University Graduate School of Medicine, Aomori, 036-8562 Japan; 6 Department of Pediatrics, Mie University Graduate School of Medicine, Tsu, Mie 514-8507 Japan

**Keywords:** Crohn's disease, Next-generation sequencing

## Abstract

A20 haploinsufficiency (HA20), a disease caused by loss-of-function *TNFAIP3* mutations, manifests various autoinflammatory and/or autoimmune symptoms. Some cases of HA20 were initially diagnosed as very early onset inflammatory bowel disease (VEO-IBD). We performed whole-exome sequencing (WES) for a Japanese girl with infantile-onset IBD and a severe perianal lesion and detected a novel de novo 119 kb microdeletion containing only *TNFAIP3* (arr[GRCh37] 6q23.3(138125829_138244816) × 1).

Very early onset inflammatory bowel disease (VEO-IBD), defined as IBD with an onset before 6 years of age, is challenging, with difficulties in diagnosis and management. Over 60 genes associated with monogenic IBD have been described worldwide with the widespread use of whole-exome sequencing (WES)^1^. Among them, *TNFAIP3* suppresses nuclear factor-kappa B, and its germline mutations lead to HA20^2^, presenting with IBD and symptoms similar to those of Behçet disease^3^. However, the diagnostic approach with genetic testing has not been well established, and the genotype−phenotype correlation in HA20 remains unclear. Here, we report a case of infantile-onset HA20 with a severe perianal lesion and a novel de novo microdeletion spanning the *TNFAIP3* gene as determined by WES and copy number variation (CNV) analysis.

The patient was an 8-month-old Japanese girl with no consanguinity or other significant family medical history. Her perinatal history was unremarkable. From the age of 3 weeks, she developed an intermittent high fever every 1−2 months that resolved without intervention. The patient’s serum C-reactive protein levels remained elevated, and abdominal computed tomography revealed hepatosplenomegaly. Screening tests previously performed at 1 month of age were not consistent with primary immunodeficiency, autoinflammatory disease, or hemophagocytic syndrome.

The patient started experiencing diarrhea more than ten times a day at 6 months of age, and active perianal fistulae appeared at the age of 7 months. She had impaired growth at 8 months of age, with a height of 63 cm (−2.5 standard deviations (SDs)) and weight of 5.3 kg (−3.0 SDs), but was not developmentally delayed. Although she was afebrile, laboratory tests indicated an abnormal condition of the autoimmune system (Supplementary Table S1). The patient had a few aphthous ulcers in the oral cavity, perianal fistulae in the right labia majora and right anterior of the anus, and anal ulcers at the anterior and dorsal sides of the anus (Fig. 1A). Esophagogastroduodenoscopy findings were unremarkable. However, ileocolonoscopy revealed aphthous ulcers throughout the colon with punched-out ulcers over the descending colon to the rectum (Fig. 1B). The terminal ileum was normal. External fistula openings ran directly to the rectal ulcers just above the dentate line. Setons were placed to drain each fistula. Although the recurrent oral aphthous ulcers, arthritis, and intestinal punched-out ulcers observed in this patient met the International or Japanese criteria for diagnosing Behçet disease, these symptoms did not fulfill the criteria for the complete or incomplete type of Behçet disease. Colonic histopathology showed mild diffuse inflammation that was primarily neutrophilic, Paneth cell metaplasia, and crypt distortion throughout the colon; further, apoptosis was observed in the sigmoid colon and descending colon. A granuloma was also noted in the hepatic flexure. Despite macroscopic inflammation, no crypt atrophy or basal plasmacytosis was present (Fig. 1C).

**Fig. 1:**
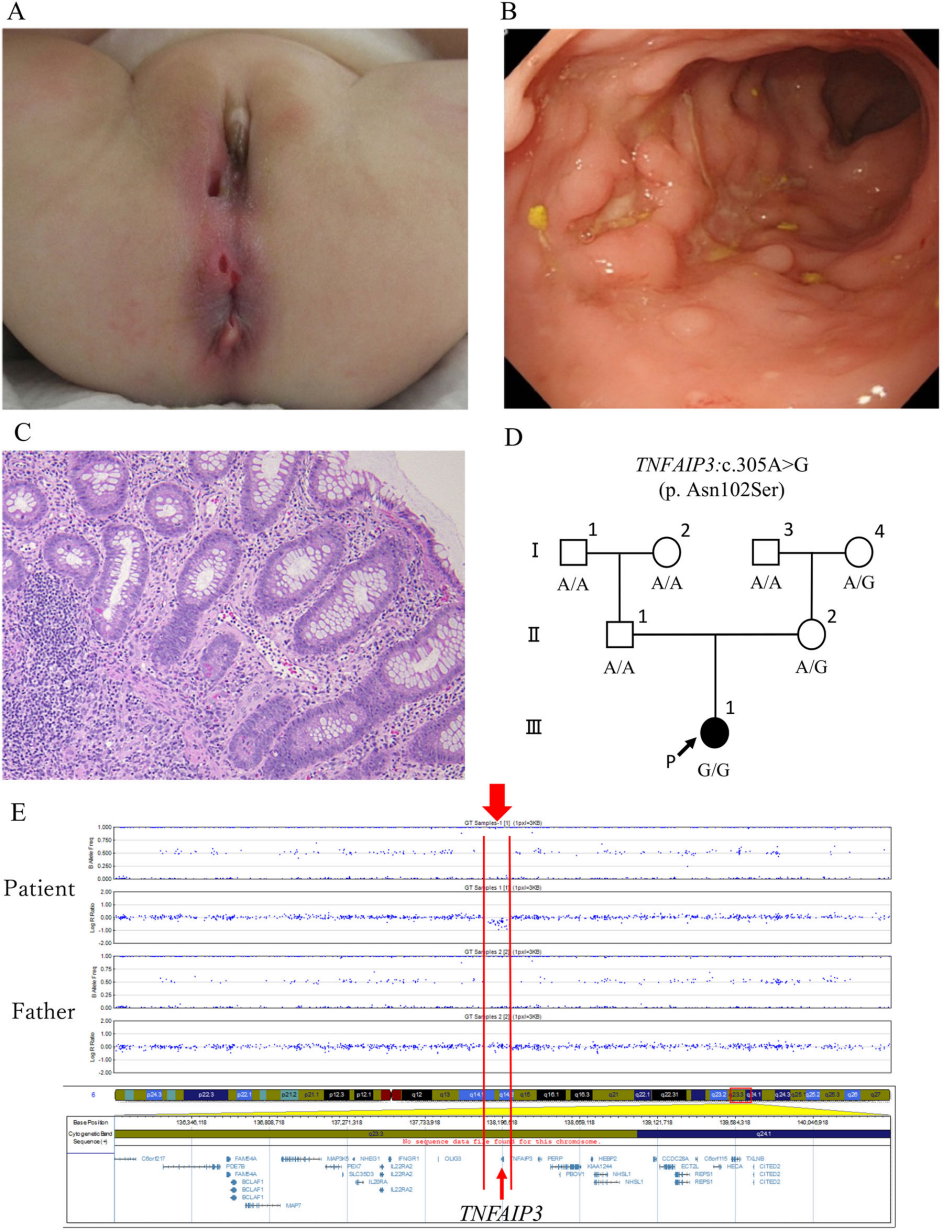
Clinical data of the patient and the TNFAIP3 genotype in her family. **A** Severe perianal fistulae. **B** Punched-out ulcers in the rectum. **C** Mild diffuse neutrophilic inflammation, Paneth cell metaplasia and apoptosis in the descending colon (hematoxylin and eosin stain; *n* ×200). **D** Pedigree chart of the *TNFAIP3* genotype (NM_001270507.2(*TNFAIP3*):c.305A>G) in the proband, her parents, and her grandparents. **E** Single-nucleotide polymorphism array of the patient and her father. Both results showed that only the patient harbored the 119 kb deletion, including only *TNFAIP3*.

The study was approved by the Institutional Review Board (IRB) of the National Center for Child Health and Development (IRB number: 926). Infantile-onset IBD with perianal lesions is a well-known manifestation of monogenic IBD, particularly in patients with IL10 signaling defects^4^; therefore, we performed WES to identify the genetic etiology of the disease as described previously^5^ after obtaining written informed consent from the patient’s family. DNA was extracted from the peripheral blood leukocytes of the patient, her parents, and her grandparents. Among the candidate variant genes, *TNFAIP3* was notable, as it had been previously reported to be associated with monogenic IBD. We could not detect any homozygous *IL10* and *IL10R* variants suspected as pathogenic in the present patient. The patient was homozygous (A/A) for *TNFAIP3* (c.305A > G; NM_001270507.2). This variant was also observed in the heterozygous state (A/G) in her mother (II-2) and is relatively common (MAF = 0.0117) in the Japanese population^6^. However, her father (II-1) was homozygous for the reference allele (G/G) (Fig. 1D), suggesting that the patient had a deletion in this region that includes *TNFAIP3*. Subsequent CNV analysis conducted with WES data using EXCAVATOR2 ^7^ revealed that the patient had only a single de novo 187 kb microdeletion within the *TNFAIP3* gene (Supplementary Fig. S1). Genome-wide single-nucleotide polymorphism array analysis of the patient and her father using the Infinium Asian Screening Array-24 v1.0 BeadChip Kit (Illumina, San Diego, CA, USA) validated that the patient had a de novo 119 kb microdeletion involving only *TNFAIP3* (arr[GRCh37] 6q23.3(138125829_138244816) × 1, accession number SCV001424911 in ClinVar) (Fig. 1E). Both WES and CNV analyses indicated that the patient had lost the paternal allele of the *TNFAIP3* gene, leading to HA20.

We detected a novel de novo microdeletion in the *TNFAIP3* gene region of a patient with infantile-onset IBD complicated by a severe perianal lesion. The present case suggests that HA20 caused by *TNFAIP3* heterozygous microdeletion should be considered when determining the cause of infantile-onset IBD with perianal lesions.

The majority of prior studies on VEO-IBD associated with HA20 have reported point mutations^3^. However, including this case, recent studies have revealed several *TNFAIP3* deletions in patients with HA20, and these are summarized in Table 1 ^8–10^. Based on a comprehensive case review^3^, nine other reports^8–16^, and the present study, five of 69 (7.2%) patients diagnosed with HA20 carried a deletion predicted to result in HA20. As approximately 7% of HA20 cases appear to be caused by deletions in *TNFAIP3*, we propose that CNV analysis should be included in the genetic testing for monogenic IBD, especially when suspecting HA20. However, Shimizu et al. reported a case possessing a very small deletion of only 5 kb in *TNFAIP3;* therefore, it is necessary to develop a smaller CNV detection algorithm using WES data to detect *TNFAIP3* microdeletion.

**Table 1:** Comparison of reported patients with HA20 and their deletion type in the literature.

Location	6q23.3	6q23.3	6q23.3	6q23.3	6q23.2−6q24.3
Range of deletion (GRCh37/hg19)	chr6:138125829_138244816	—	chr6:138192201−138428412	chr6:138192201−138428412	chr6:134387945−147518246
Size of deletion	119 kb	5 kb	236 kb	236 kb	13.13 Mb
Involved genes	*TNFAIP3*	*TNFAIP3* (exons 2−3)	*TNFAIP3, PERP*	*TNFAIP3, PERP*	*SGK1, ALDH8A1 HBS1L, MYB, AHI1, PDE7B, MTFR2, BCLAF1, MAP7, MAP3K5, PEX7, SLC35D3, IL20RA, IL22RA2, IFNGR1, OLIG3, TNFAIP3, PERP, ARFGEF3, PBOV1, HEBP2, NHSL1, GVQW2, CCDC28A, ECT2L, REPS1, ABRACL, HECA, TXLNB, CITED2, NMBR, VTA1, ADGRG6, HIVEP2, AIG1, ADAT2, PEX3, FUCA2, PHACTR2, LTV1, ZC2HC1B, PLAGL1, SF3B5, STX11, UTRN, EPM2A, FBXO30, SHPRH, GRM1, RAB32, ADGB*
Gender	Female	Male	Female	Female	Male
Initial symptoms	Periodic fever	Recurrent oral and perianal ulcer	Periodic fever	Oral ulcer	Upper respiratory infections, gastroenteritis, febrile episodes
Symptom onset	3 weeks	6 years	2 months	1 year	1 year
Age at diagnosis	8 months	9 years	11 years	16 years	12 years
Phenotype	Periodic fever, abdominal pain, diarrhea, bloody stools, failure to thrive, digestive ulcers, perianal fistulae	Recurrent episodes of abdominal colic with fever, headache, vomiting, and oral/perianal ulcers	Periodic fever, abdominal pain, diarrhea, bloody stools, weight loss, digestive ulcers, acute anterior uveitis, pharyngalgia, enlarged tonsil	Oral ulcer, folliculitis, abdominal pain, thyroiditis	Neutrophilic dermatosis, growth and psychomotor delay, oral aphthae, diarrhea, perianal ulcers
Treatment	Colchicine, 5-aminosalicylic acid, surgical placement of setons	Adalimumab	Colchicine, cimetidine, mesalazine, PSL, NSAIDs, MTX, corticosteroid eye drops	Levothyroxine	Etanercept
References	This report	(Shimizu et al.^10^)	(Tsuchida et al.^8^)	(Tsuchida et al.^8^)	(Franco-Jarava et al.^9^)

*NSAIDs* nonsteroidal anti-inflammatory drugs, *MTX* methotrexate, *PSL* prednisolone.

For her infantile-onset perianal lesion, seton drainages were required for 1 year after their placement. Although infantile-onset IBD with perianal lesions is a well-known manifestation of monogenic IBD, particularly in patients with IL10 signaling defects^4^, this patient did not have any homozygous *IL10* and *IL10R* mutations despite low serum IL10 levels. Some HA20 cases show perianal lesions, but their severity is variable, and cases with severe perianal fistula, as in this case, are very rare. As VEO-IBD cases undergoing WES are increasing^8,12–15^, more cases including the deletion type will be accumulated in the future. It is also expected that the phenotype-genotype correlation will be elucidated. Overall, HA20 should be included as a differential diagnosis for infantile-onset IBD with perianal lesions, and CNV analysis in addition to WES should be considered to evaluate the *TNFAIP3* deletion.

In conclusion, patients with HA20 caused by TNFAIP3 microdeletions can present infantile-onset IBD with severe perianal lesions. New genomic technologies to analyze microdeletions should be used to further investigate previously undiagnosed cases of monogenic IBD.

## Supplementary information

Supplemental TableS1

Supplemental FigureS1

## Data Availability

The relevant data from this Data Report are hosted at the Human Genome Variation Database at 10.6084/m9.figshare.hgv.2903.
