# Supplementary material for: Novel TNFAIP3 microdeletion in a girl with infantile-onset inflammatory bowel disease complicated by a severe perianal lesion
Source: Hum Genome Var. 2021 Jan 14;8:1. doi: 10.1038/s41439-020-00128-4 (PMC7809258; doi:10.1038/s41439-020-00128-4)
Supplement: Supplementary file 1 — Supplemental TableS1 [file 41439_2020_128_MOESM1_ESM.docx]

**Supplemental Table S1**

|  |  |  |
| --- | --- | --- |
| white blood cell counts |  | 18,060 cells/μL |
| C-reactive protein |  | 2.6 mg/dL (normal, 0-0.3 mg/dL) |
| erythrocyte sedimentation rate |  | 48 mm/60 min (normal, 3-15 mm/hour) |
|  |  |  |
| interleukin (IL) -6 |  | 19 pg/mL (normal, <0.5 pg/mL) |
| IL-10 |  | <2 pg/ml (normal, <5 pg/mL) |
| soluble tumor necrosis factor receptor (sTNFR) -1 |  | 4,040 pg/mL (normal, 484-1,407 pg/mL) |
| sTNFR-2 |  | 13,900 pg/mL (normal, 829-2,262 pg/mL) |
| IL-18 |  | 1,520 pg/mL (normal, <500 pg/mL) |
| Serum human leukocyte antigen typing | B51 | negative |
|  | A26 | negative |
| Anti-nuclear antibody (2months) | Peripheral | < 40× (normal, < 40×) |
|  | Homogeneous | < 40× (normal, < 40×) |
|  | Speckled | 80× (normal, < 40×) |
|  | Nucleolar | < 40× (normal, < 40×) |
|  | Centromere/Discrete-Speckled | < 40× (normal, < 40×) |
| rheumatoid factor |  | 5IU/ml (normal, < 15IU/ml) |
|  |  |  |
| Immunological workup, including serum immunoglobulins, lymphocyte blastic transformation, neutrophil sterilizing function and flow cytometry of peripheral blood, |  | no apparent abnormalities |
|  |  |  |
| T-SPOT |  | negative |
| EBV-IgM |  | negative |
| EBV-IgG |  | negative |
| C7-HRP |  | negative |
|  |  |  |
| Stool culture |  | negative for enteric infection |
|  |  |  |

**Supplemental Table 1**

Clinical laboratory test results of the patient at 8 months of age.
