# Supplementary material for: Novel TNFAIP3 microdeletion in a girl with infantile-onset inflammatory bowel disease complicated by a severe perianal lesion
Source: Hum Genome Var. 2021 Jan 14;8:1. doi: 10.1038/s41439-020-00128-4 (PMC7809258; doi:10.1038/s41439-020-00128-4)
Supplement: Supplementary file 2 — Supplemental FigureS1 [file 41439_2020_128_MOESM2_ESM.docx]

**Supplemental Figure S1**


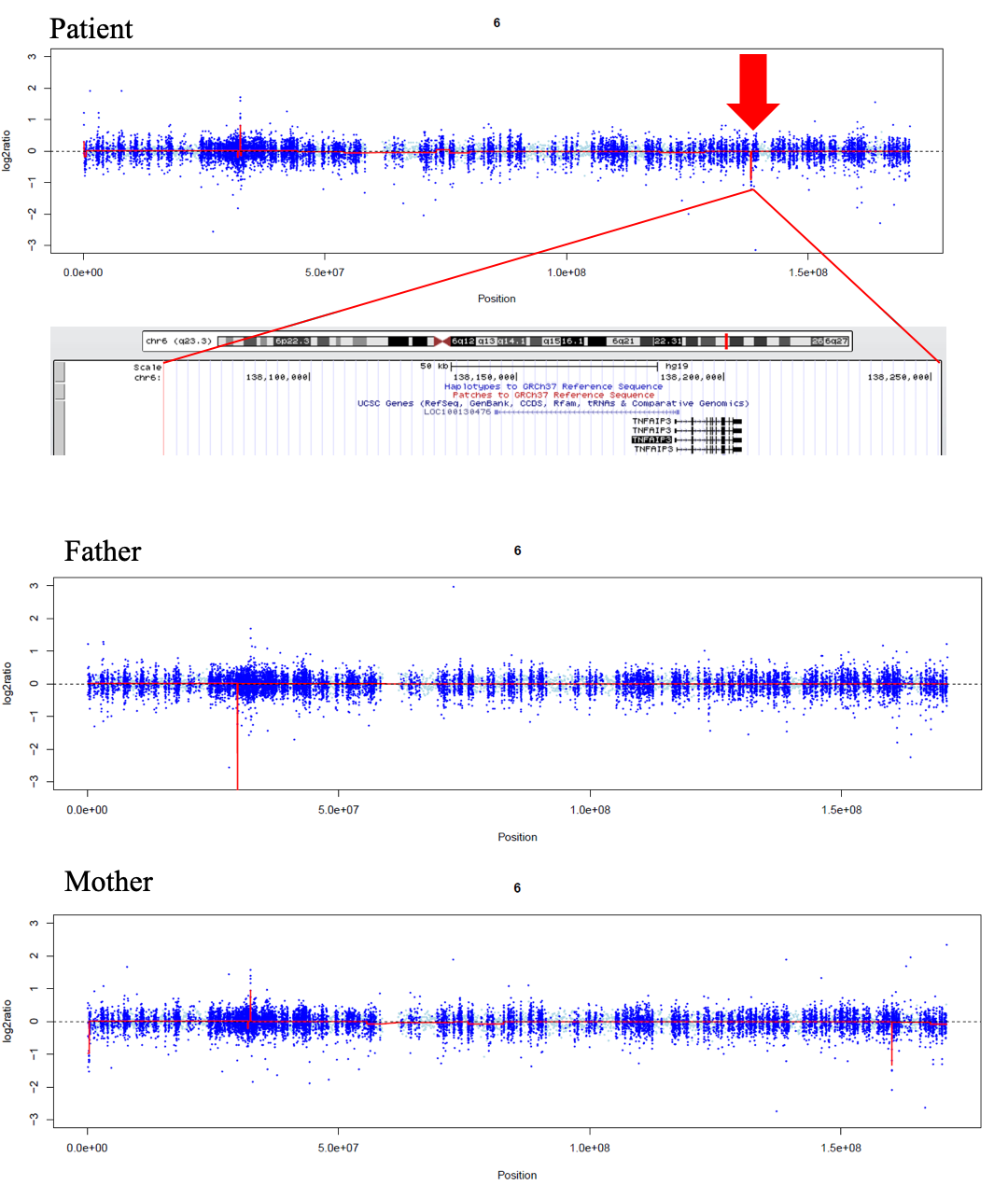


**Supplemental Fig 1**

The results of CNV analysis of the patient and her parents by EXCAVATOR2.
